# Supplementary material for: Learning curve and surgical outcome of robotic assisted colorectal surgery with ERAS program
Source: Sci Rep. 2022 Nov 29;12:20566. doi: 10.1038/s41598-022-24665-w (PMC9709162; doi:10.1038/s41598-022-24665-w)
Supplement: Supplementary file 1 — Supplementary Information 1. [file 41598_2022_24665_MOESM1_ESM.zip › LSC Raw data-Ñ[▒K/116/H_ñw╝╨░O▒Kñσ.pdf]

正式摘要(電子簽章：0000002104163 86)

W65 / W65 ☐英文標題(注意:無英文姓名-請補病人基本檔!) ☐【讀取】憑證

臺中榮民總醫院

出院病歷摘要

轉入號 : 轉出號 :

[redacted]

**(8)**流水編號	
**(9)**入院日期	110/01/31 (2021) 胃腸肝膽科 W65 - 022
**(10)**轉科日期	110/02/04 (2021) - 直腸外科
**(11)**出院日期	110/02/12 (2021) 住院天數計 12 日

診斷

- 入院:Multiple liver metastasis R/O proximal colon cancer with liver metastasis.
- 出院:1. Rectal cancer with multiple liver and lung metastasis, cT4N1M1c(4.3cm mass)
  s/p laparoscopic LAR on 2021/2/5 (for obstructing sign)
  2. HBV(HbsAg+, anti Hbe+, HbeAg-)
  - consult GI for HBV medications before chemotherapy
  3.HP infection

主訴

- right upper quandrant abdominal dull pain for 2 weeks

病史

- This 68 y/o male farmer with hx of
  1.HBV, chronic hepatitis
  2.DM, under medication
  3.HTN, under medication
  he was admitted due to RUQ abdmen pain in 01/17. Accompanied symptom was poor appetite. Besides, according to the patient, pencil-like stool and tenesmus was noted for 1-2 months. And he went to LMD first. PE revealed A1-A2 mild tenderness. Body weight loss was also noted(82kg->76kg in recent 6 months) . Abdomen sono revealed multiple hepatic tumor. Therefore, he was referred to our OPD for further survey. CXR revealed RLL one nudule superimprose with right diaphragm. KUB revealed much stool. Tumor marker showed CEA:2099, CA-199:460, AFP:1.01. HBsAg:(+). Abdomen sono revealed bilateral liver lobes multiple hyper/hypoechoic lesion. There was no fever, no diaphoresis, no decreased appetitie, no dysuria, no family related cancer history. He had smoking habits for 1PPD for 30 yrs, quit 20 yrs. Social drinking. No betal nuts chewing. Due to liver metastasis can not be excluded. He is admitted for lver biopsy and abdomen CT for further survey.

體檢發現

- KUB: local ileus and
  - General appearance: A well-nourished person, with acute ill looking. Consciousness: Alert Pupil size: 2.5/2.5 mm Light reflex:
    - Normal light reflex

    Vital signs:

date	體溫(BT)	脈搏(P)	呼吸(R)	血壓(BP)	身高(BH)	體重(BW)	BMI
20210131	37.3℃	83次/分	16次/分	147/92mmHg	165.3cm	76.8kg	28.1

  - Skin
    - Normal skin turgor , No jaundice , No cyanosis , No rash , No wound / ulcer / scar
  - Lymphadenopathy
    - No lymphadenopathy
  - HEENT
    - Head: No shape deformity , No hair change , Normal lip
    - Eyes: ＊Conjunctiva: Normal , ＊Sclera: Not icteric , ＊Cornea: Clear , ＊

- Eyelids: Normal
    - Ears: No deformity
    - Nose: Normal appearance
    - Mouth and throat: No oral mucosa change , No throat injection , No tonsil enlargement
- Neck
    - No thyroid enlargement , Neck: Jugular vein
- Chest
    - Inspection: Normal and symmetric respiratory movement , No deformity , Normal breast , No spider angiomata
    - Palpation: No palpable mass , No tender point , No vocal fremitus , No subcutaneous crepitance
    - no abnormal dullness or hyperresonance
    - Auscultation: No rales , No rhonchi , No wheezing , Chest / Auscultation / Stridor No stridor , No peripheral brochial sound , No friction rub
- Heart
    - Inspection: No visible apical impulse
    - Palpation: PMI over 5 th rib , No thrill , No heave
    - Auscultation: Regular heart beats , No S3 , No S4 , No murmur , No pericardial friction rub
- Abdomen
    - Inspection: No distension , No striae
    - Auscultation: Normal bowel sound ,
        - No bruit
    - Palpation: No hepatomegaly , No tenderness , No rebounding pain , No palpable mass , No splenomegaly
    - Percussion: No shifting dullness , No central tympanic
- Genito-anus patient refuse
- Back and spine
    - No deformity , No knocking pain
- Extremities
    - No deformity , No clubbing finger , No pitting edema , Normal joint , Normal peripheral pulse , No acrocyanosis
    - No palm erythema , No muscular tenderness
- Neurological examination
    - No mental status change
    - Normal cranial nerve
    - Normal meninges
    - flexor response
    - Normal motor
    - Normal sensory
    - Normal cerebellum
    - Normal Stance & Gait

手術日期及方法

- 手術日期及方法20210205 1. Laparoscopic low anterior resection, end-to-end anastomosis, Endo-GIA 60-4.1mm*2, CDH 33mm.
  Bilateral doughnut: intact, air tight test: done, no air leak.
  2. High ligation (+) above the left colic artery.
  3.r/o peritoneal seeding, s/p resection and sent to pathologic prove
  4. Cut margin: proximal 10 cm, distal 2 cm.
  5. Take down of splenic flexure: (-).
  6. A 12mm penrose drain placed in the pelvis.

住院治療經過

- After admission, we arranged series of survey. tumor marker showed elevated CEA and CA 199. abdominal CT showed colon cancer with multiple liver and lung metastasis, cT4N1M2(4.3cm mass). Further colonoscopy showed rectal cancer with partial lumen obstruction. UGI scope on 2/3 reported reflux esophagitis and antral erosions s/p biopsy for H.p plate test. H.P plate test showed positive. HBV infection(HbsAg+, anti Hbe+, HbeAg-) was noted. We are still pending for HBV viral load. Due to rectal cacer with multiple liver and lung

- metastasism, CRS was consulted and we took over the patient, we had explain to the patient, which operation was needed due to obstruction sign, and we offer the patient opions as colonstomy or AR, they choose AR, laparoscopic AR was done on 2/5. After the operation, NG was removed on 2/6 and try water. Bland one diet was tried on 2/8, however due to distended abd, NPO on 2/9. Foley was removed on 2/9. The patient had flattus on 2/10, bland two diet was add on 2/10. Penrose was revmoved on 2/11. Under stable condition, the patient was discharge with OPD follow up.

合併症

一般檢查

特殊檢查

病理報告

其它

出院時情況

- 3.改門診治療

出院指示

藥名	劑量	劑量單位	用法	頻次	數量	數量單位	現況	天數	單位
MOSAD tab 5mg	1	TAB	PO	TID	21	TAB	TH	7	天
Through tab	1	TAB	PO	QN	7	TAB	TH	7	天
Acetal tab 500mg	1	TAB	PO	Q6HPRN	28	TAB	TH	7	天

出院計劃

主治醫師 2062F 蔣鋒帆 / 住院醫師 2332G 林志安

正式摘要(電子簽章：**000000210416386)**

W65 / W65

doctorRank:B
